# Multiple, not single, recipient muscle tendon transfers produce well-coordinated thumb-tip movement in lateral pinch grasp: a simulation study with application to restoration of improved grasp after tetraplegia

**DOI:** 10.3389/fbioe.2025.1532424

**Published:** 2025-04-09

**Authors:** Cole D. Smith, Joseph D. Towles

**Affiliations:** Department of Engineering, Swarthmore College, Swarthmore, PA, United States

**Keywords:** tendon transfer surgeries, spinal cord injury, thumb muscles, lateral pinch movement, multi-muscle control

## Abstract

**Introduction:**

Tendon transfer surgeries that engage the flexor pollicis longus (FPL) muscle are commonly performed to enable lateral pinch grasp in persons with tetraplegia. Functional outcomes, however, have been mixed. This may be the case, in part, because the FPL produces hyperflexion at the interphalangeal (IP) joint and radial deviation at the carpometacarpal (CMC) joint. Therefore, the goal of this simulation study was to investigate whether small groups of muscles could produce movement with less IP joint hyperflexion and CMC joint ab/adduction than the FPL produces during lateral pinch grasp.

**Methods:**

We adapted a published, open-source computational musculoskeletal model of the hand to simulate lateral pinch grasp movement. A forward dynamics simulation approach was used to drive the thumb, with 27 muscle groups being considered, from an extended posture to a flexed posture to make contact with the side of the index finger. We calculated CMC joint ab/adduction deviation from the flexion–extension plane and IP joint flexion in the plane that all muscle groups produced and compared those joint angle movements to those of the FPL when it alone drove the thumb.

**Results:**

Of the 27 simulations, three muscle groups, each consisting of three or four muscles, generated lower IP joint flexion and CMC joint ab/adduction compared with the FPL.

**Conclusion:**

This study points to the potential of novel, multiple recipient muscle tendon transfer surgeries to outperform the current standard of care to restore lateral pinch grasp following tetraplegia.

## 1 Introduction

**FIGURE 1:**
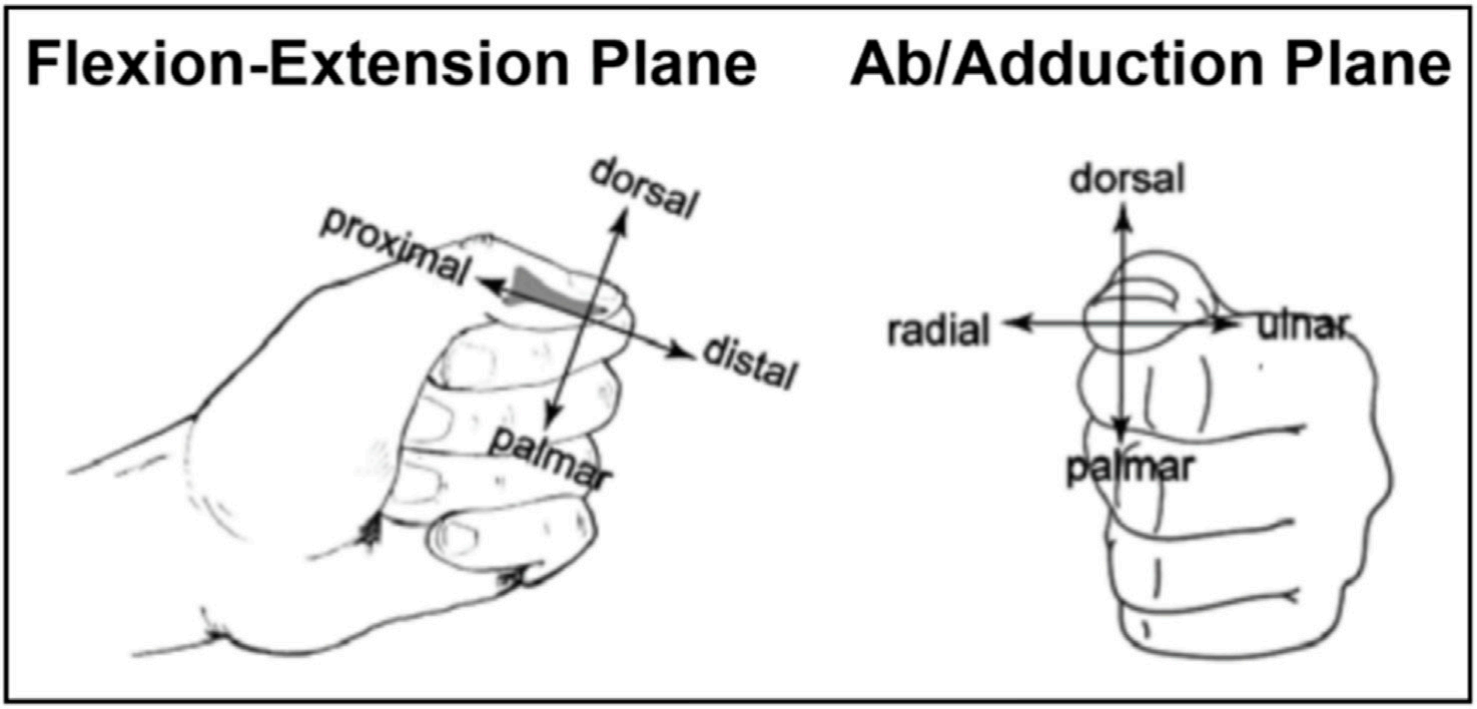
Lateral Pinch Grasp. Lateral pinch grasp is defined as contact between the thumb and the side of the index finger. Pictured is lateral pinch grasp as viewed from the planes of flexion-extension and ab/adduction with distal phalanx anatomic reference frame directions shown in each plane.

Loss of hand function severely affects the quality of life of up to 135,000 persons around the world who suffer a cervical spinal cord injury (SCI) annually (Lee et al., 2014; Spinal Cord Injury Facts and Figures at a Glance, n. d.). Tendon transfer surgeries that engage the flexor pollicis longus (FPL) muscle, either indirectly or directly, are commonly performed to enable lateral pinch grasp (Fox et al., 2018; Hentz, 2002; Revol et al., 2002). Lateral pinch grasp is characterized by contact between the thumb pad and the lateral aspect of the index finger (Figure 1). It is one of the simplest grasp modalities that allow for accomplishing activities of daily living (ADLs) (Cutkosky and Howe, 1990). Tendon transfers that restore lateral pinch grasp attempt to create good and strong grasp contact between the thumb and index finger. Good contact is characterized by a large portion of the thumb pad making contact with the lateral index finger and strong contact is characterized by a grasp contact pinch force that is primarily directed at or perpendicular to the index finger and is of a magnitude that can accomplish activities of daily living (Smaby et al., 2004). The Moberg Procedure (Moberg, 1975) was the first successful tendon transfer surgical procedure that restored lateral pinch grasp in persons with C5-C6 level SCI (i.e., SCI injury between the 5th and 6th cervical vertebrae). Moberg demonstrated that lateral pinch grasp could be restored in 1 of 2 ways: either by attaching the donor muscle, the brachioradialis (BR), to the paralyzed recipient muscle, the extensor carpi radialis brevis (ECRB), or attaching the BR to the FPL. In the first case, restored wrist extension causes the FPL tendon and finger flexor tendons to develop tension, thus producing lateral pinch grasp through the tenodesis effect (indirect engagement of the FPL) (Hentz, 2002). In the second case, where the ECRB is not paralyzed, the BR actuates the FPL directly to flex the thumb against the lateral index finger to produce lateral pinch grasp (direct engagement of the FPL). In the years that have followed the introduction of the Moberg procedure, surgical approaches–aimed at restoring lateral pinch grasp in the C5-C6 level SCI patient population–have not significantly deviated from the chief principles that Moberg put forth (Fox et al., 2018; Hentz, 2002; Revol et al., 2002).

The FPL is the most convenient muscle choice to ultimately engage for this procedure because it causes the least amount of radial–ulnar deviation and is the only thumb muscle that flexes all three of the interphalangeal (IP), metacarpophalangeal (MP), and carpometacarpal (CMC) joints (Paul Smutz et al., 1998). Functional outcomes, however, have been mixed: maximum post-surgical pinch strength has differed up to 10-fold among patients and has been as low as tenths of a pound (Johanson et al., 2016; Waters et al., 1985). This outcome is possibly due to the oblique direction of the FPL’s endpoint force, which contributes to weak pinch force and a tendency for thumb-tip slip during grasp contact (Buchholz et al., 1988; Murray et al., 2017; Towles, 2023; Towles et al., 2008). From a joint movement perspective, the FPL tends to cause hyperflexion of the IP joint due to the ratio of the FPL’s flexion moment arms at the IP, MP, and CMC joints (Paul Smutz et al., 1998). Interphalangeal joint hyperflexion diminishes the likelihood of good grasp contact in which the main portion of the thumbpad makes contact with the lateral aspect of the index finger. In this case, lateral pinch contact is not made at all or is made between the very distal portion of the thumb-tip, which, over time, induces callus formation on the thumb-tip. Stabilization of the IP joint by split FPL tenodesis (Van Heest et al., 1999) or Steinman pin Hentz et al. (1983) has been used to reduce or eliminate, respectively, the FPL’s tendency to hyperflex the IP joint and therefore enable thumbpad contact with the lateral index finger. From the perspective of the thumb-tip force that the FPL produces, joint stabilization improves its oblique directional nature, but only slightly (Towles et al., 2004). Taken together, the FPL’s endpoint force production and joint movement characteristics are less than ideal for the muscle to be the lone driver of the thumb.

**FIGURE 2:**
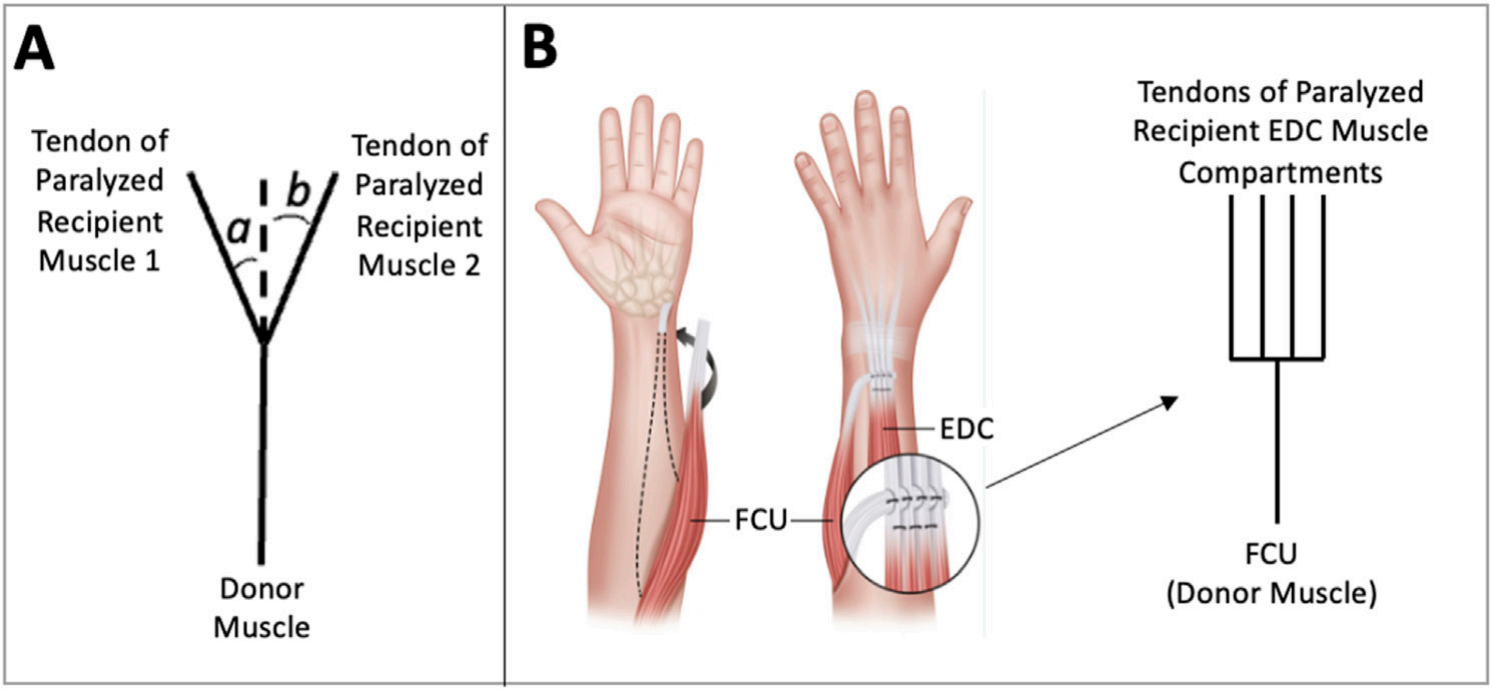
Multiple Recipient Muscle Tendon Transfer Diagrams. In this tendon transfer arrangement, the donor muscle is attached to multiple paralyzed recipient muscles. Two are pictured here. **(A)** In this diagram, the donor muscle force is transferred to the tendons of paralyzed muscles 1 and 2. Note that the line of action of the force that the donor muscle transfers to the paralyzed recipients is not aligned with lines of actions of the recipient muscles. The proportion of force transferred to each recipient muscle tendon depends on the values of angles *a* and *b*. **(B)** In this illustration, the flexor carpi ulnari-to-extensor digitorum communis (FCU to EDC) tendon transfer is presented and is used to restore finger extension after radial nerve injury (Sankaran et al., 2015). In this surgery, the donor muscle force of FCU is transferred equally to the four tendons of the paralyzed EDC muscle compartments.

More generally, the surgical outcomes of restoring lateral pinch grasp after cervical SCI or tetraplegia may be mixed because tendon transfer surgeries have not historically been designed with a biomechanics-based understanding of the endpoint velocities and endpoint forces that muscles generate during a grasping task. Previous studies that modeled multiple recipient muscle tendon transfer surgeries, in which the non-paralyzed donor muscle is attached to multiple paralyzed recipient muscles (as in Figure 2A), have also shown that small groups of muscles produced more palmarly directed forces (i.e., forces that better promote thumb-tip stability during pinch) than the current standard of care using the FPL alone (Towles, 2023). In this study, we make a distinction between a multi-insertion site tendon transfer in which the donor muscle connects to multiple recipient muscles in such a way that the line of action of the pull force of the donor muscle is not aligned with the force transmitted to the group of recipient muscles (Figure 2A) and a multi-insertion site tendon transfer in which that alignment in forces exists (Figure 2B). In this study, we are focused on the tendon transfer described in Figure 2A.

A preliminary execution of a mathematical model determined that only muscle combinations (e.g., multiple recipient muscle tendon transfers as depicted in Figure 2A), and not individual muscles, have the capacity for the desired thumb-tip movement and that these muscle combinations should include the FPL. In a previous simulation study, we found that four groups of two muscles, 10 groups of three muscles, and 13 groups of four muscles produced well-directed endpoint forces during lateral pinch in the flexed thumb (Towles, 2023). It is unknown, however, if these muscle combinations produce ideal lateral pinch movement from an extended to a flexed thumb posture.

The goal of this study was to investigate the joint movement characteristics of such muscle combinations in comparison to the current standard of care. Specifically, we wanted to know whether they could produce movement of the thumb primarily in the flexion–extension (FE) plane as the thumb moved from an extended posture to a flexed posture, making contact with the lateral aspect of the index finger to simulate a lateral pinch movement. We hypothesized that at least one of the muscle combinations being considered would produce a more ideal movement pattern than the movement that FPL produces alone.

## 2 Materials and methods

To investigate whether groups of thumb muscles could flex the thumb primarily in the FE plane from an extended thumb posture to a flexed one, we used a previously developed musculoskeletal model of the hand in the OpenSim framework. Our approach entailed adapting the model of the thumb (McFarland et al., 2023) and implementing forward dynamic simulations of the thumb—driven by the muscle groups of interest—to facilitate lateral pinch from a wide-grip posture to a narrow one as the thumbpad made contact with the lateral aspect of the index finger. The biomechanical model of the wrist and hand is available as an open-source model, incorporating 22 rigid bodies using mass and inertial properties from previous experimental work (Binder-Markey et al., 2019). Properties of the muscles of the thumb, index finger, and wrist were obtained from previous literature as specified in previously validated models (Kamper et al., 2002; Domalain et al., 2010; Knutson et al., 2000; Li et al., 2006). Parameters defined in those validated models were replicated for the remaining muscles (Binder-Markey et al., 2019; Wohlman and Murray, 2013; Saul et al., 2015). The model has been validated for kinetic simulations of grip and lateral pinch strength using experimental force production data (McFarland et al., 2023; Valero-Cuevas et al., 2003). Kinematic simulations of hand opening, aligned with muscle activation data, and simulations of tenodesis grasp and release displayed the expected coupled motion between the wrist and fingers (McFarland et al., 2023).

### 2.1 Adaptation of the musculoskeletal model

**FIGURE 3:**
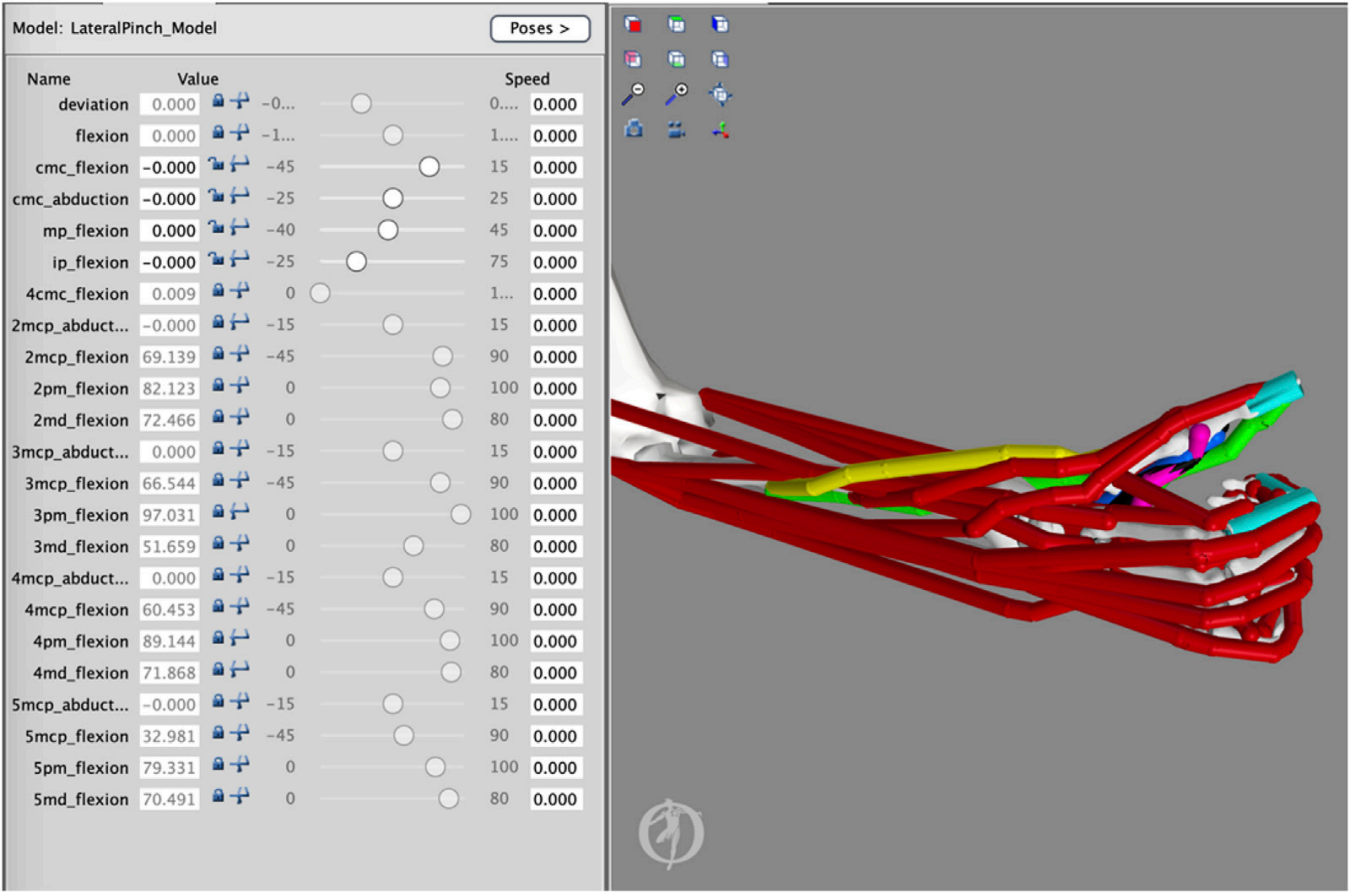
Adapted Thumb Model. The starting extended posture of the thumb was characterized by 0° of flexion and ab/adduction at the carpometacarpal (CMC) joint, 0° of flexion at the metacarpophalangeal (MP) joint, and 0° of flexion at the interphalangeal (IP) joint. Massless bodies attached to the thumb-tip and index finger were used to detect contact between the thumb and index finger.

The movement of the thumb-tip, driven by specific muscle combinations, was analyzed to determine whether any sets of muscles were capable of producing ideal lateral pinch movement patterns of the thumb-tip throughout the FE plane, characterized by lower peak radial or ulnar deviation and IP joint flexion than that produced by FPL alone. These simulations were carried out using an adapted version of a previously developed hand and wrist model in OpenSim (Version 4.3) (McFarland et al., 2023; Delp et al., 2007). Briefly, the model consisted of FE and ab/adduction degrees of freedom (DOFs) at the CMC joint, an FE DOF at the MP joint, an FE DOF at the IP joint, and FE and ab/adduction DOFs at the wrist. The model also included nine thumb muscles. Some thumb muscles have multiple names. To compare this study to the previous study (Towles, 2023) on which this one is based, we will use thumb muscle names from that study and parenthetically note the alternative names used in the model (McFarland et al., 2023) where they apply. The names of the nine thumb muscles were the FPL, the ulnar head of the flexor pollicis brevis (FPBu) (model: transverse head of the adductor pollicis), the radial head of the flexor pollicis brevis (FPBr) (model: flexor pollicis brevis), the adductor pollicis (ADP) (model: oblique head of the adductor pollicis), the opponens pollicis (OPP), the abductor pollicis brevis (APB), the abductor pollicis longus (APL), the extensor pollicis longus (EPL), and the extensor pollicis brevis (EPB). The model was adapted both in terms of the implementation of the elastic foundation used and the starting posture. Specifically, elastic foundation components were introduced as massless cylinders overlaying the thumb-tip and the proximal, middle, and distal phalanges of the index finger. These cylinders allowed the skin of the fingers to be approximated by massless rigid bodies and the contact force between these bodies to be calculated (Figure 3). Contact parameters, representing the skin of the thumb and index finger, were taken from studies that validated the model around pinch and grip strength exercises (McFarland et al., 2023; Li et al., 2011). The thumb was then set to an extended position defined as 0° of flexion and abduction at the CMC joint, 0° of flexion at the MP joint, and 0° of flexion at the IP joint (Figure 3). As illustrated in Figure 3, neutral flexion at the IP joint occurred when the long axes of the proximal and distal phalanges aligned and neutral flexion at the MP joint occurred when the long axes of the metacarpal and the proximal phalanx aligned. As described by Paul Smutz et al. (1998), neutral flexion at the CMC joint occurs when the thumb rests on the side of the index finger in a lateral pinch posture. The other four digits were flexed to match the hand posture in a previous lateral pinch force study, and the wrist was set to 0° of joint flexion and 0° of joint abduction (Figure 3) (McFarland et al., 2023; Nichols et al., 2017).

We then used this adapted model orientation to explore both muscle group–driven and FPL-driven thumb flexion from an extended thumb posture. The 27 muscle groups we investigated are consistent with those in a related previous study (Towles, 2023) and are listed in Table 1. At the end of thumb flexion, the thumb made contact with the index finger as determined by the point at which the elastic foundation force deviated from zero.

**TABLE 1:** Thumb muscle groups.

Groups of two muscles	Groups of three muscles	Groups of four muscles
1 2	1 2 6	1 2 3 6
1 3	1 6 7	1 2 5 6
1 4	1 4 8	1 3 6 7
3 5	1 2 3	1 4 5 6
	1 2 5	1 2 3 8
	1 3 7	1 3 7 8
	1 3 4	1 2 3 7
	1 3 5	1 2 3 5
	1 3 9	1 2 4 5
	1 4 5	1 3 5 7
		1 3 4 5
		1 3 5 9
		1 4 5 7

Muscle groups consisted of two, three, or four muscles. The numbers represent the following muscles: (1) flexor pollicis longus (FPL), (2) flexor pollicis brevis-radial head (FPBr), (3) flexor pollicis brevis-ulnar head (FPBu), (4) adductor pollicis (ADP), (5) abductor pollicis longus (APL), (6) extensor pollicis longus (EPL), (7) abductor pollicis brevis (APB), (8) extensor pollicis brevis (EPB), and (9) opponens pollicis (OPP).

### 2.2 Model simulation

**FIGURE 4:**
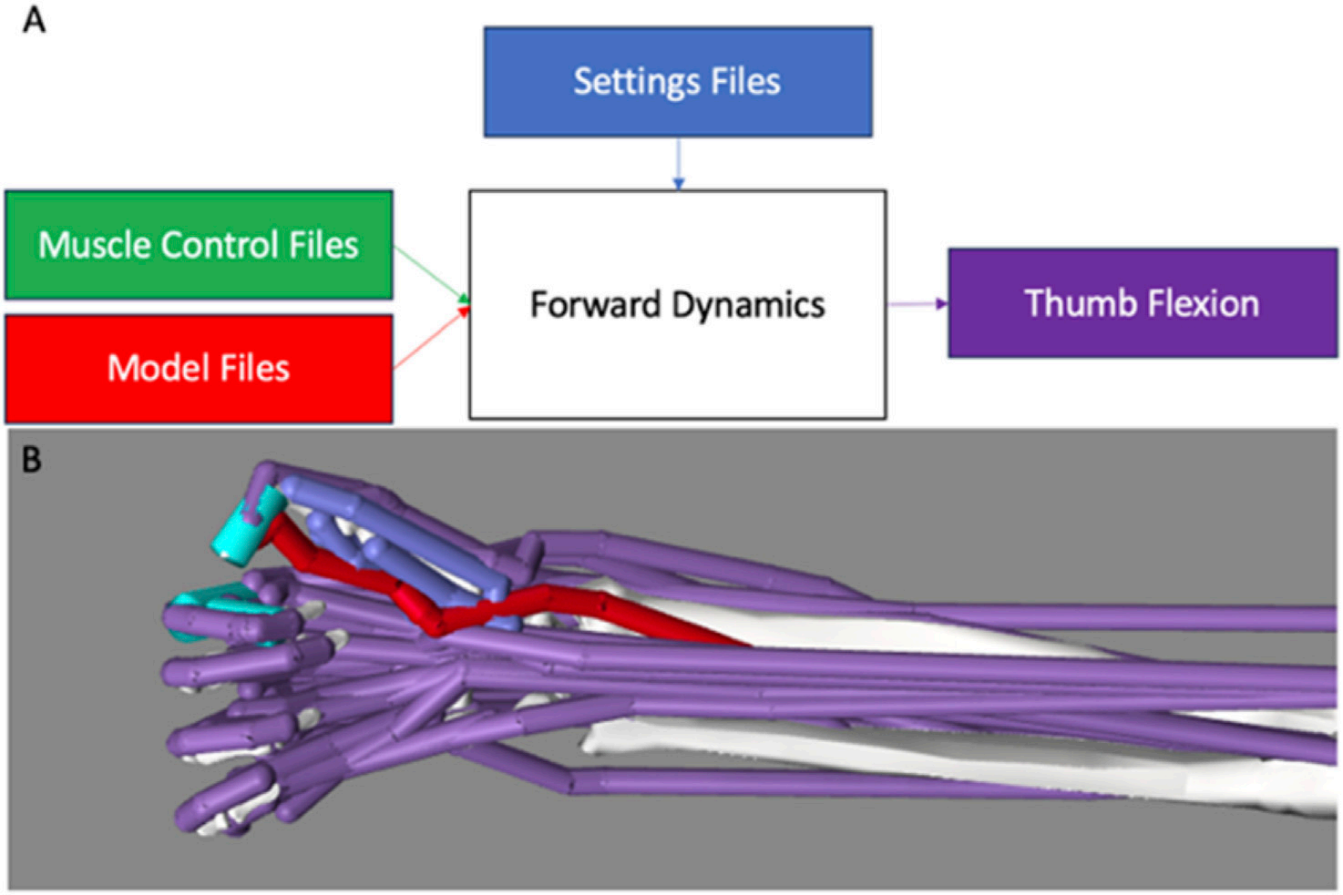
Overview of the OpenSim Forward Dynamics Framework. **(A)** The adapted model (red) and. sto muscle control files (green) were loaded into the Forward Dynamics tool. A settings .xml file (blue) was then loaded to set the runtime of the model, set directories, and define the results to be printed following completion of the simulation (purple). **(B)** During forward dynamics, only activated muscles (red) drive movement; the remaining non-activated muscles (purple) act passively. The FPL was active in this example.

To implement muscle group–driven thumb flexion in OpenSim, the forward dynamics routine was used (Figure 4). In the forward dynamics routine, activation files were created for each muscle combination and FPL such that only the FPL or the muscles in the combination of interest were activated at 100% to actuate the thumb. When muscles were activated, they exerted their effect simultaneously on the thumb rather than in a coordinated fashion. From this, it also follows that muscles likely exerted different and various levels of muscle force throughout the range of motion of lateral pinch movement. During the simulation, the finger joints were fixed in their flexed postures and the wrist joint was fixed in its neutral posture. Twenty-eight simulations were carried out using OpenSim’s Forward Dynamics tool to investigate how the FPL and each combination of muscles impacted thumb-tip movement during the execution of lateral pinch grasp from an extended thumb posture to a flexed thumb posture. During this movement, we quantified IP joint flexion and CMC ab/adduction. Simulated thumb movements were integrated over 0.15 s, in line with other movement simulations used in the validation of the model (McFarland et al., 2023; Nichols et al., 2017), allowing the thumb to make contact with the index finger and come to rest.

### 2.3 Simulation analysis

During lateral pinch movement, maximum IP joint flexion, maximum CMC joint abduction, and maximum CMC joint adduction were calculated relative to the starting joint positions (using a MATLAB script). CMC joint adduction and abduction values were combined to determine the range of motion outside the FE plane. Our analysis sought to determine muscle combinations that generated less IP joint flexion and CMC joint ab/adduction compared with those produced by FPL alone. Maximum IP joint flexion and CMC joint range of motion were reported for each combination and ranked to determine which combinations minimized IP joint flexion, CMC joint ab/adduction, and both characteristics of the 28 simulations. Combinations that outperformed FPL in both respects were reported as combinations that could produce more favorable thumb-tip movement compared with the FPL throughout the plane of lateral pinch movement.

## 3 Results

**FIGURE 5:**
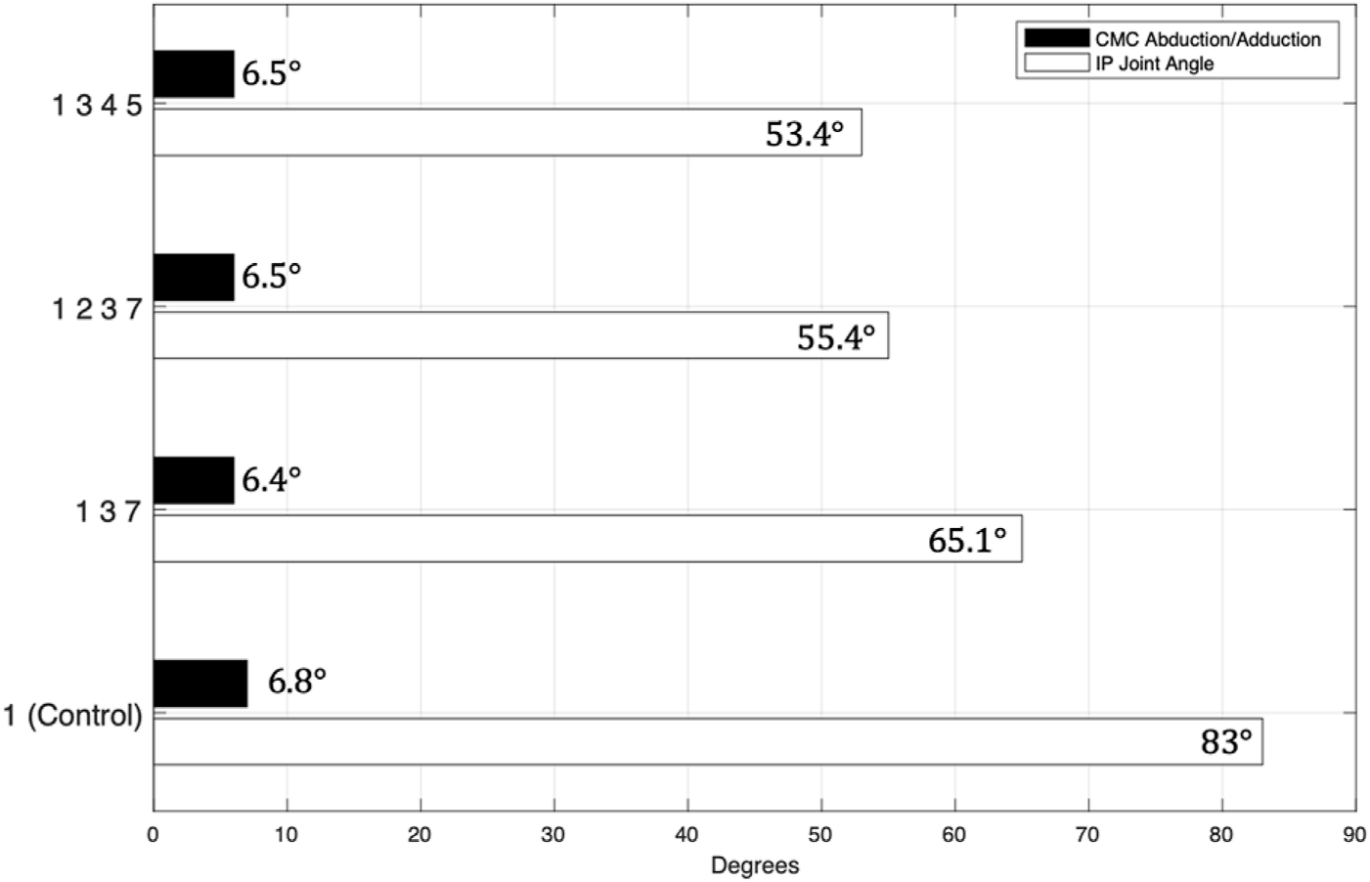
Muscle Combinations that Produced More Favorable Thumb-Tip Movement Compared with FPL Alone in Both Planes. The numbers represent the following muscles: (1) flexor pollicis longus (FPL), (2) flexor pollicis brevis-radial head (FPBr), (3) flexor pollicis brevis-ulnar head (FPBu), (4) adductor pollicis (ADP), (5) abductor pollicis longus (APL), (6) extensor pollicis longus (EPL), (7) abductor pollicis brevis (APB), (8) extensor pollicis brevis (EPB), and (9) opponens pollicis (OPP).

**FIGURE 6:**
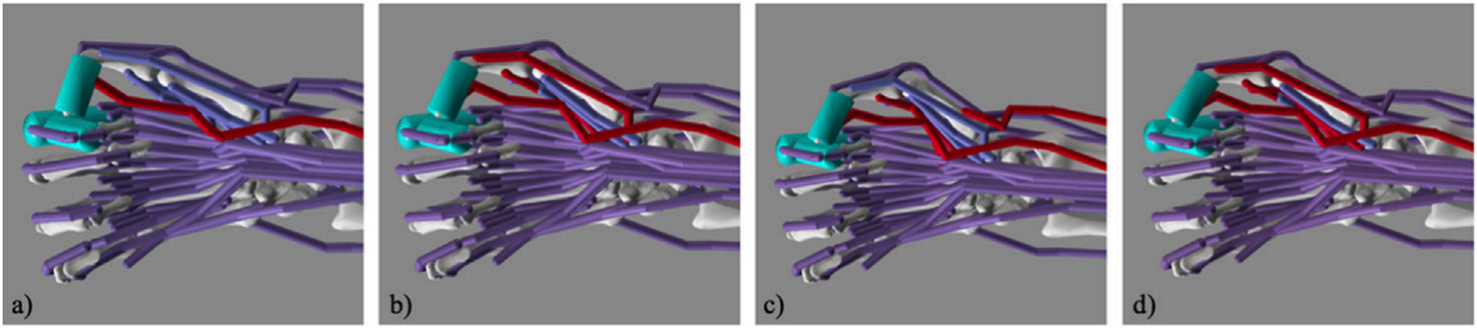
Simulated Lateral Pinch Involving the FPL Only and Various Muscle Groups. Images of the **(a)** flexor pollicis longus (FPL); **(b)** FPL, flexor pollicis brevis-ulnar head (FPBu), and abductor pollicis brevis (APB); **(c)** FPL, FPBu, adductor pollicis (ADP), and abductor pollicis longus (APL); and **(d)** FPL, flexor pollicis brevis-radial head (FPBr), FPBu, and APB at contact in the simulation.

As we hypothesized, at least one muscle group produced less CMC joint ab/adduction and less IP joint flexion compared with the FPL alone. Specifically, 11% (3 of 27) of the muscle groups generated less IP joint flexion than the FPL (i.e., less than 83°) and less CMC joint ab/adduction than FPL (i.e., less than 6.7°) (Figures 5, 6). Specifically, FPL, FPBu, ADP, and APL produced 53° of IP joint flexion; FPL, FPBr, FPBu, and APB, 55°; and FPL, FPBu, and APB, 65° (Figures 5, 6). All three combinations contain FPL and FPBu.

The model achieved pinch in all 28 simulations, yielding an average maximum IP joint flexion of approximately 70° and an average range of motion of approximately 12° for abduction or adduction at the CMC joint. Flexion at the IP joint ranged from 41° (FPBu + APL) to 90° (EPL + FPL + APB), and ab/adduction at the CMC joint ranged from 6° (FPL + EPB + FPBu + APB) to 23° (EPL + FPL + FPBr + FPBu) (Table 2).

**TABLE 2:** Forward dynamics simulation results.

Combination	Max IP joint angle (^o^)	IP rank	Total CMC ab/adduction (^o^)	CMC rank
Control (FPL Alone)				
1	83.0	21	6.8	5
Groups of Two Muscles				
1 2	72.0	16	7.3	6
1 3	60.0	8	9.9	12
1 4	67.0	14	10.1	13
3 5	41.1	1	14.0	20
Groups of Three Muscles				
1 2 6	88.3	26	22.3	27
1 6 7	89.7	28	19.9	25
1 4 8	88.7	27	9.6	10
1 2 3	51.6	3	10.3	14
1 2 5	80.9	20	19.1	24
**1 3 7**	**65.1**	**11**	**6.4**	**2**
1 3 4	50.6	2	11.7	17
1 3 5	66.3	13	11.2	15
1 3 9	55.1	5	8.5	7
1 4 5	74.2	17	11.4	16
Groups of Four Muscles				
1 2 3 6	65.8	12	22.8	28
1 2 5 6	85.7	24	12.8	19
1 3 6 7	79.7	19	21.7	26
1 4 5 6	84.0	23	15.4	23
1 2 3 8	84.0	22	9.5	9
1 3 7 8	87.5	25	6.2	1
**1 2 3 7**	**55.4**	**6**	**6.5**	**4**
1 2 3 5	55.9	7	9.6	11
1 2 4 5	61.7	10	9.3	8
1 3 5 7	67.0	15	14.2	21
**1 3 4 5**	**53.4**	**4**	**6.5**	**3**
1 3 5 9	60.9	9	12.4	18
1 4 5 7	75.2	18	14.6	22
Average	69.6		12.1	

Maximum IP joint flexion, flexion rank; CMC joint range of motion, and range of motion rank are presented for the 28 simulations. The numbers in the muscle group combinations represent the following muscles: (1) FPL, (2) FPBr, (3) FPBu, (4) ADP, (5) APL, (6) EPL, (7) APB, (8) EPB, and (9) OPP.

Bold values designate groups of muscles that performed better than FPL alone in both lower max IP joint angle and total CMC ab/adduction.

The muscle groups outperformed the FPL mostly in IP joint flexion and, to a lesser degree, in CMC joint ab/adduction. Of the 27 muscle group simulations, 74% (20 of 27) of the muscle groups flexed the IP joint less than the FPL did alone, and 15% (4 of 27) of the muscle groups generated less CMC joint ab/adduction movement than the FPL did alone. Both of these movements contributed to reduced IP joint hyperflexion and out-of-plane movement of the thumb during lateral pinch.

## 4 Discussion

The primary goal of this work was to show that, in a musculoskeletal model (McFarland et al., 2023), combinations of small groups of muscles can generate improved thumb-tip movement patterns from an extended thumb posture to a flexed posture to achieve lateral pinch. This study was motivated by the need to find small groups of muscles that have both favorable endpoint force (Towles, 2023) and endpoint movement characteristics that outperform those of the FPL to restore lateral pinch grasp in persons with a cervical SCI. As we hypothesized, multiple groups of muscles produced a more ideal lateral pinch movement pattern than the movement that FPL produces alone. Three such muscle groups produced both less ab/adduction at the CMC joint and less flexion at the IP joint: (1) FPL, FPBu, and APB; (2) FPL, FPBr, FPBu, and APB; and (3) FPL, FPBu, ADP, and APL. *To the best of our knowledge, this is the first simulation study to show the potential of small groups of thumb muscles to restore desirable lateral pinch movement.* Furthermore, this study is significant because it is the first to explicitly consider lateral pinch movement throughout its range of motion from a biomechanical perspective, and the results have the potential to improve lateral pinch grasp restorative surgeries in persons with cervical SCI.

The results of the study can be explained by musculoskeletal mechanics. Experimental measurements of ab/adduction moment arm data for thumb muscles throughout the plane of FE do not exist. For reference, they exist for a single flexed thumb posture (Paul Smutz et al., 1998). Notwithstanding, the finding that out-of-plane deviations at the CMC joint during thumb flexion from an extended thumb posture to a flexed posture were comparable in three muscle groups (Figure 4) could be explained by similar group-wide average muscle length variations that are approximately the same as that of the FPL. The 20 of 27 muscle groups that outperformed the FPL (Table 2) by generating less IP joint flexion primarily consisted of some combination of the FPL plus uniarticular and/or biarticular muscles that flex the CMC joint only (uniarticular) or the CMC and MP joints (biarticular) (Table 2). One such group was the FPL combined with the ADP and OPP, a biarticular muscle and a uniarticular muscle, respectively. In dynamic simulations, both uniarticular muscles that flex the CMC joint only and biarticular muscles that flex both the CMC and MP joints can extend the IP joint. This non-intuitive action at the IP joint can occur because of complex dynamic coupling in a multi-joint musculoskeletal system, which may also explain why these muscle groups produced less IP joint flexion than the FPL produced alone.

Reduced IP joint hyperflexion and out-of-plane movement of the thumb during lateral pinch can lead to a healthier and higher-quality grasp. Reduced hyperflexion of the IP joint reduces the risk of callus formation on the thumb tip and creates greater thumb surface contact during grasping. The latter improves the quality of the contact during grasping because thumbpad surface torques can be passively resisted if need be (Murray et al., 2017). Thumb-tip torques can be resisted because of frictional interactions between skin surface area and the surface of the object being contacted. Moreover, reduced out-of-plane movement results in a greater potential for ideal contact between the thumb and the object being grasped (i.e., the thumb landing on the central portion of the grasped object). This thumb positioning improves the grasp quality because the thumb is more likely than otherwise to maintain contact with the object in the presence of any grasp disturbances.

The three muscle groups that resulted in improved lateral pinch movement characteristics contained both the FPL and the FPBu. The finding that the FPBu is common to these groups likely points to its broadly complementary endpoint velocity characteristics that support more favorable lateral pinch movement than the FPL produces alone. This finding further supports the idea that intrinsic muscles have the potential to improve lateral pinch grasp characteristics (e.g., endpoint force and velocity directions) (Towles, 2023; Towles et al., 2008). As we previously stated, we think that a small number of paralyzed muscles simultaneously driven by a donor muscle can produce more favorably directed endpoint movement compared with the FPL’s during lateral pinch grasp, leading to improved grasp contact with consequent implications for grasp force production (Murray et al., 2017). The idea that only muscle combinations (e.g., multiple recipient muscle tendon transfers)—not individual muscles—have the capacity for the desired thumb-tip movement naturally follows from the understanding that multi-muscle control is required for accurate positioning of the thumb and, ultimately, strong and stable grasps. However, a general tenet of tendon transfer surgery is to attach one donor muscle to only one paralyzed recipient muscle (i.e., single recipient muscle tendon transfer) and to do so such that there is a straight line path between the tendon of the donor muscle and that of the recipient muscle (Hentz et al., 1983). A common exception to this tenet is when a straight line path between the tendon of the donor muscle and that of multiple tendons of recipient muscle compartments (e.g., FCU to EDC tendon transfer, Figure 2B) can be achieved. We believe that this tenet exists because of the difficulty of surgically planning, by inspection, the control of multiple recipient muscles by one donor muscle when the donor muscle and recipient muscles are not connected along a straight line path. Computational musculoskeletal and cadaveric surgical simulation tools have not historically been used to plan lateral pinch tendon transfer surgeries. We believe that both can be used to investigate the potential of multiple recipient muscle tendon transfer surgeries.

As far as we know, this simulation study is the first to apply a musculoskeletal modeling approach to investigate the potential of small groups of thumb muscles to restore lateral pinch movement throughout the FE plane following cervical SCI. This work highlights the possibility of employing multiple recipient muscle tendon transfer surgeries to that end. Investigating the surgical feasibility of engaging multiple recipient muscles, including intrinsic muscles, in tendon transfer surgery and determining how to appropriately coordinate the donor muscle force across the recipient muscles are critical steps to consider in future computational modeling, cadaver-based and clinical experiments.

## Data Availability

The original contributions presented in the study are included in the article/supplementary material, further inquiries can be directed to the corresponding author.

## References

[B1] Binder-Markey B. I. Dewald J. P. A. Murray W. M. (2019). The biomechanical basis of the claw finger deformity: a computational simulation study. J. Hand Surg. Am. 44 (9), 751–761. 10.1016/j.jhsa.2019.05.007 31248678 PMC6718315

[B2] Buchholz B. Frederick L. J. Armstrong T. J. (1988). An investigation of human palmar skin friction and the effects of materials, pinch force and moisture. Ergonomics 31 (3), 317–325. 10.1080/00140138808966676 3383835

[B3] Cutkosky M. R. Howe R. D. (1990). “Human grasp choice and robotic grasp analysis,” in Dexterous robot hands. Editors Venkataraman S. Iberall T. (New York: Springer), 5–31. 10.1007/978-1-4613-8974-3_1

[B4] Delp S. L. Anderson F. C. Arnold A. S. Loan P. Habib A. John C. T. (2007). OpenSim: open-source software to create and analyze dynamic simulations of movement. IEEE Trans. Biomed. Eng. 54 (11), 1940–1950. 10.1109/TBME.2007.901024 18018689

[B5] Domalain M. Vigouroux L. Berton E. (2010). Determination of passive moment-angle relationships at the trapeziometacarpal joint. J. biomechanical Eng. 132 (7), 071009. 10.1115/1.4001397 20590287

[B6] Fox I. K. Miller A. K. Curtin C. M. (2018). Nerve and tendon transfer surgery in cervical spinal cord injury: individualized choices to optimize function. Top. Spinal Cord Inj. Rehabilitation 24 (3), 275–287. 10.1310/sci2403-275 PMC6037328 29997430

[B7] Hentz V. R. (2002). Surgical strategy: matching the patient with the procedure. Hand Clin. 18, 503–518. 10.1016/S0749-0712(02)00030-6 12474600

[B8] Hentz V. R. Brown M. Keoshian L. A. (1983). Upper limb reconstruction in quadriplegia: functional assessment and proposed treatment modifications. J. Hand Surg. 8 (2), 119–131. 10.1016/S0363-5023(83)80001-X 6833719

[B9] Johanson M. E. Dairaghi C. A. Hentz V. R. (2016). Evaluation of a task-based intervention after tendon transfer to restore lateral pinch. Archives Phys. Med. Rehabilitation 97 (6), S144–S153. 10.1016/j.apmr.2015.12.032 27233589

[B10] Kamper D. G. George Hornby T. Rymer W. Z. (2002). Extrinsic flexor muscles generate concurrent flexion of all three finger joints. J. biomechanics 35 (12), 1581–1589. 10.1016/s0021-9290(02)00229-4 12445611

[B11] Knutson J. S. Kilgore K. L. Mansour J. M. Crago P. E. (2000). Intrinsic and extrinsic contributions to the passive moment at the metacarpophalangeal joint. J. biomechanics 33 (12), 1675–1681. 10.1016/S0021-9290(00)00159-7 11006392

[B12] Lee B. B. Cripps R. A. Fitzharris M. Wing P. C. (2014). The global map for traumatic spinal cord injury epidemiology: update 2011, global incidence rate. Spinal Cord. 52 (2), 110–116. 10.1038/sc.2012.158 23439068

[B13] Li C. Guan G. Reif R. Huang Z. Wang R. K. (2011). Determining elastic properties of skin by measuring surface waves from an impulse mechanical stimulus using phase-sensitive optical coherence tomography. J. R. Soc. Interface 9 (70), 831–841. 10.1098/rsif.2011.0583 22048946 PMC3306653

[B14] Li Z. M. Davis G. Gustafson N. P. Goitz R. J. (2006). A robot-assisted study of intrinsic muscle regulation on proximal interphalangeal joint stiffness by varying metacarpophalangeal joint position. J. Orthop. Res. 24, 407–415. 10.1002/jor.20046 16479575

[B15] McFarland D. C. Binder-Markey B. I. Nichols J. A. Wohlman S. J. de Bruin M. Murray W. M. (2023). A musculoskeletal model of the hand and wrist capable of simulating functional tasks. IEEE Trans. Biomed. Eng. 70 (5), 1424–1435. 10.1109/TBME.2022.3217722 36301780 PMC10650739

[B16] Moberg E. (1975). Surgical treatment for absent single-hand grip and elbow extension in quadriplegia. Principles and preliminary experience. J. Bone Jt. Surg. 57 (2), 196–206. 10.2106/00004623-197557020-00012 1112846

[B17] Murray R. M. Li Z. Sastry S. S. (2017). A mathematical introduction to robotic manipulation. 1st ed. Boca Raton, FL, United States CRC Press. Available online at: https://www.taylorfrancis.com/books/9781351469791.

[B18] Nichols J. A. Bednar M. S. Wohlman S. J. Murray W. M. (2017). Connecting the wrist to the hand: a simulation study exploring changes in thumb-tip endpoint force following wrist surgery. J. Biomechanics 58, 97–104. 10.1016/j.jbiomech.2017.04.024 PMC5536109 28552412

[B19] Paul Smutz W. Kongsayreepong A. Hughes R. E. Niebur G. Cooney W. P. An K.-N. (1998). Mechanical advantage of the thumb muscles. J. Biomechanics 31 (6), 565–570. 10.1016/S0021-9290(98)00043-8 9755041

[B20] Revol M. Cormerais A. Laffont I. Pedelucq J.-P. Dizien O. Servant J.-M. (2002). Tendon transfers as applied to tetraplegia. Hand Clin. 18 (3), 423–439. 10.1016/S0749-0712(02)00028-8 12474594

[B21] Sankaran A. Thora A. Arora S. Dhal A. (2015). Single tendon transfer of the flexor carpi ulnaris for high radial nerve injury. J. Orthop. Surg. (Hong Kong) 23 (3), 345–348. 10.1177/230949901502300318 26715715

[B22] Saul K. R. Hu X. Goehler C. M. Vidt M. E. Daly M. Velisar A. (2015). Benchmarking of dynamic simulation predictions in two software platforms using an upper limb musculoskeletal model. Comput. methods biomechanics Biomed. Eng. 18 (13), 1445–1458. 10.1080/10255842.2014.916698 PMC4282829 24995410

[B23] Smaby N. Johanson M. E. Baker B. Kenny D. E. Murray W. M. Hentz V. R. (2004). Identification of key pinch forces required to complete functional tasks. J. Rehabilitation Res. Dev. 41 (2), 215–224. 10.1682/JRRD.2004.02.0215 15558375

[B24] Towles J. D. (2023). Measurement of the three-dimensional muscle endpoint forces in the extended thumb and its application to determining muscle combinations that enable lateral pinch force production throughout the plane of flexion-extension. Annu. Int. Conf. IEEE Eng. Med. Biol. Soc. 2023, 1–5. 10.1109/EMBC40787.2023.10340817 38083628

[B25] Towles J. D. Hentz V. R. Murray W. M. (2008). Use of intrinsic thumb muscles may help to improve lateral pinch function restored by tendon transfer. Clin. Biomech. 23 (4), 387–394. 10.1016/j.clinbiomech.2007.11.008 18180085

[B26] Towles J. D. Murray W. M. Hentz V. R. (2004). The effect of percutaneous pin fixation of the interphalangeal joint on the thumb-tip force produced by the flexor pollicis longus: a cadaver study. J. Hand Surg. 29 (6), 1056–1062. 10.1016/j.jhsa.2004.07.005 15576215

[B27] Valero-Cuevas F. J. Johanson M. E. Towles J. D. (2003). Towards a realistic biomechanical model of the thumb: the choice of kinematic description may be more critical than the solution method or the variability/uncertainty of musculoskeletal parameters. J. biomechanics 36 (7), 1019–1030. 10.1016/s0021-9290(03)00061-7 12757811

[B28] Van Heest A. Hanson D. Lee J. Wentdorf F. House J. (1999). Split flexor pollicus longus tendon transfer for stabilization of the thumb interphalangeal joint: a cadaveric and clinical study. J. Hand Surg. 24 (6), 1303–1310. 10.1053/jhsu.1999.1303 10584958

[B29] Waters R. Moore K. R. Graboff S. R. Paris K. (1985). Brachioradialis to flexor pollicis longus tendon transfer for active lateral pinch in the tetraplegic. J. Hand Surg. 10 (3), 385–391. 10.1016/S0363-5023(85)80040-X 3998421

[B30] Wohlman S. J. Murray W. M. (2013). Bridging the gap between cadaveric and *in vivo* experiments: a biomechanical model evaluating thumb-tip endpoint forces. J. Biomech. 46 (5), 1014–1020. 10.1016/j.jbiomech.2012.10.044 23332233 PMC3627365

